# Evaluation of Mortality and Morbidity According to Initial Symptoms in COVID-19 Patients Using Clinical Epidemiologic Data from the Korea Centers for Disease Control & Prevention

**DOI:** 10.3390/medicina57070688

**Published:** 2021-07-06

**Authors:** So Young Kim, Dae Myoung Yoo, Chanyang Min, Joo-Hee Kim, Hyo Geun Choi

**Affiliations:** 1 CHA Bundang Medical Center, Department of Otorhinolaryngology-Head & Neck Surgery, CHA University, Seongnam 13496, Korea; sossi81@hanmail.net; 2 Hallym Data Science Laboratory, Hallym University College of Medicine, Anyang 14068, Korea; ydm1285@naver.com (D.M.Y.); joicemin@naver.com (C.M.); 3 Graduate School of Public Health, Seoul National University, Seoul 08826, Korea; 4 Division of Pulmonary, Allergy, and Critical Care Medicine Department of Medicine, Hallym University Sacred Heart Hospital, Hallym University College of Medicine, Anyang 14068, Korea; luxjhee@gmail.com; 5 Department of Otorhinolaryngology-Head & Neck Surgery, Hallym University College of Medicine, Anyang 14068, Korea

**Keywords:** symptom assessment, morbidity, mortality, covid-19, case-control studies, cohort studies

## Abstract

*Background and Objectives:* This study aimed to investigate whether initial symptoms of COVID-19 are associated with mortality and morbidity. *Materials and Methods*: The data of 5628 laboratory-confirmed COVID-19 patients were collected by the Korea Centers for Disease Control and Prevention. The maximum level of morbidity during hospital admission was classified as mild or severe, and patient mortality was recorded. Clinical symptoms were categorized as respiratory, gastrointestinal, general, and neurologic symptoms. The hazard ratios (HRs) for clinical symptoms associated with mortality were analyzed using the Cox proportional hazards model. The odds ratios (ORs) for clinical symptoms associated with morbidity were analyzed using the logistic regression model. *Results*: Of the included COVID-19 patients, 15.4% (808/5253) were classified as having severe morbidity. Morbidity was related to the clinical symptoms of cough, sputum, shortness of breath, vomiting/nausea, diarrhea, fever, and altered mental status or confusion. According to the symptom categories, respiratory and general symptoms were related to high morbidity (OR = 1.41, 95% CI = 1.30–1.53, *p* < 0.001 for respiratory symptom and OR = 1.37, 95% CI = 1.18–1.59, *p* < 0.001 for general symptom). Mortality was associated with the clinical symptoms of shortness of breath, fever, and altered mental status or confusion. Among the symptom categories, respiratory symptoms were associated with a 1.17-fold increased HR for mortality (95% CI = 1.04–1.32, *p* = 0.008). *Conclusions*: Initial respiratory symptoms were related to high morbidity and mortality in COVID-19 patients.

## 1. Introduction

Coronavirus disease 2019 (COVID-19) has been spreading worldwide since December 2019 [1]. Although more than 80% of COVID-19 patients experience mild respiratory symptoms, approximately 13.8% of COVID-19 patients suffer from severe symptoms, and approximately 3.1% of patients worldwide have critical disease courses [2]. Severe acute respiratory syndrome coronavirus 2 (SARS-CoV-2) primarily impacts CD4+ and CD8+ T lymphocytes by interfering with their activities, including the production of interferon-γ [3]. Severe COVID-19 patients often present with high levels of inflammatory markers such as C-reactive protein, interleukin-2R (IL-2R), IL-6, IL-10, and tumor necrosis factor-α as well as symptoms of dyspnea [3]. The viral pathogen of COVID-19, SARS-CoV-2, has high infectivity, which warrants the active quarantining of patients [4]. A few studies reported that the highest infectivity occurred at the presymptomatic or initial symptom stages; this occurred in approximately 44% (95% confidence interval [95% CI] = 25–69%) of infector–infectee transmission pairs [4]. The peak viral load also coincided with initial symptom onset [4,5,6]. Therefore, it can be presumed that the initial presentation of COVID-19 could provide crucial clues regarding the severity and/or prognosis of COVID-19.

Previous studies have investigated the correlations of symptoms of COVID-19 with disease severity, prognosis, and viral load [5,7,8,9]. A high viral load has been suggested to be related to severe disease [5,8]; however, some previous studies reported no associations between symptomatic or asymptomatic disease and viral load [7,10]. The site of virus sampling, such as the throat, nasopharyngeal wall, or serum, and the time of viral examination may influence these variable outcomes. For COVID-19, some clinical symptoms have been reported to be associated with the severity of disease. For instance, diarrhea, an extrapulmonary symptom, was suggested to be related to mild disease [11]. In addition, the symptoms of dyspnea and anosmia were related to a relatively low viral load [7]. On the other hand, dyspnea was also related to an increased risk of septic shock in COVID-19 patients [11]. These discrepancies could be partially explained by the variable study population and the disease stage at the time of symptom evaluation. Few studies have evaluated multiple symptoms, including respiratory, gastrointestinal, and neurologic symptoms, of COVID-19 in relation to disease morbidity or mortality.

If initial symptoms affect the clinical course of COVID-19, an estimation of the types of symptoms and the degree of their effects would be vital information for managing COVID-19 patients. This study hypothesized that the initial symptoms of COVID-19 might serve as a critical predictor of morbidity and mortality in COVID-19 patients. To test this hypothesis, all initial respiratory, gastrointestinal, general, and neurologic symptoms were analyzed for their associations with morbidity of and mortality due to COVID-19. First, each symptom was analyzed for its relation with COVID-19-related morbidity and mortality. Then, multiple symptoms were categorized as respiratory, gastrointestinal, general, and neurologic symptoms, and the contributions of each symptom category in COVID-19-related morbidity and mortality were evaluated.

## 2. Materials and Methods

### 2.1. Ethics

The ethics committee of Hallym University (2020-07-032) approved this study. Written informed consent was waived by the Institutional Review Board. All analyses adhered to the guidelines and regulations of the ethics committee of Hallym University.

### 2.2. Study Population and Participant Selection

The clinical and epidemiological data of all confirmed COVID-19 patients who were released from isolation before 30 April 2020, were collected. The data were collected by the Korea Centers for Disease Control and Prevention. COVID-19 was confirmed by PCR, and patients were released from isolation after complete recovery. Confirmed patients without symptoms were considered to be completely recovered if the PCR test result was negative twice in a row with a 24-h interval 7 days after the definitive diagnosis. Confirmed patients with symptoms were considered to be completely recovered if their fever resolved without antipyretic drugs, if the clinical manifestations had improved and if the PCR test result was negative twice in a row with a 24-h interval 7 days after the definitive diagnosis.

**Figure 1:**
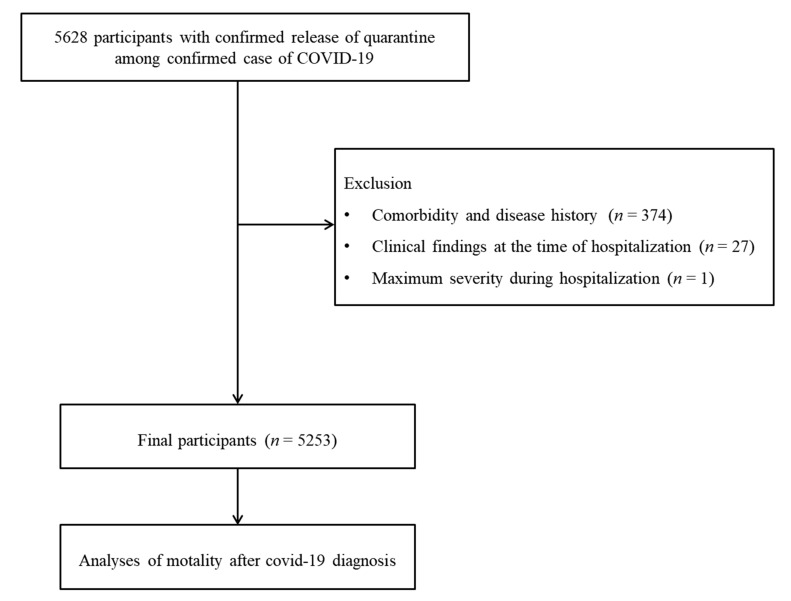
Schematic illustration of the participant selection process that was used in the present study. Of a total of 5628 confirmed COVID-19 patients, 5253 patients were enrolled.

Patients who did not have accompanying symptoms or past medical histories were excluded (*n* = 374). Patients who did not have clinical symptoms at the time of hospitalization were excluded (*n* = 27). Patients who did not experience the maximum level of morbidity during hospitalization were excluded (*n* = 1). Finally, 5253 patients were selected (Figure 1).

### 2.3. Exposure (Clinical Symptoms)

Patients were screened for the following clinical symptoms at the time of hospitalization to guide COVID-19 treatment: cough, sputum production, sore throat, rhinorrhea, shortness of breath, vomiting or nausea, diarrhea, fever, myalgia, fatigue or malaise, headache, and altered mental status or confusion.

Clinical symptoms were categorized and scored as follows: respiratory symptoms were scored as 1–5 points (cough, sputum production, sore throat, rhinorrhea, and shortness of breath), gastrointestinal symptoms were scored as 1–2 points (vomiting or nausea, diarrhea), general symptoms were scored as 1–3 points (fever, myalgia, fatigue or malaise), and neurologic symptoms were scored as 1–2 points (headache, mental status alteration or confusion). The score was calculated and considered a continuous variable to calculate the overall effects of these symptom groups.

### 2.4. Outcome (Mortality)

During follow-up from 19 February 2020 to 30 April 2020, all death events were recorded.

### 2.5. Outcome (Morbidity)

The maximum level of morbidity during admission was classified as follows: no limited activity, limited activity but no oxygen supplementation, oxygen supplementation with a nasal cannula, oxygen supplementation with a facial mask, noninvasive ventilation, invasive ventilation, multiorgan failure/extracorporeal membrane oxygenation (ECMO), or death. The maximum level of morbidity during admission was also categorized as follows: mild (no limited activity, limited activity but no oxygen supplementation), and severe (oxygen supplementation with a nasal cannula, oxygen supplementation with a facial mask, noninvasive ventilation, invasive ventilation, multiorgan failure/ECMO, death).

### 2.6. Covariates

Age groups were divided into 10-year intervals: 0–9, 10–19, 20–29…, and 80+ years old (total of nine age groups). Systolic blood pressure was divided into five categories: <120, 120–129, 130–139, 140–159, and ≥150 mmHg. Diastolic blood pressure was divided into four categories: <80, 80–89, 90–99, and ≥100 mmHg. Obesity was measured using BMI (body mass index, kg/m^2^). BMI was divided into five categories: <18.5 (underweight), ≥18.5 to <23 (normal), ≥23 to <25 (overweight), ≥25 to <30 (obese I), and ≥30 (obese II). Heart rate (rate/min) and body temperature (°C) were directly measured. Missing systolic blood pressure and diastolic blood pressure (*n* = 33 (0.6%)) values were replaced with 120–129 and 80–89 mmHg, respectively, and temperature (*n* = 39 (0.7%)) and heart rate (*n* = 129 (0.3%)) values were replaced with the mean values of each variable from the included patients. Missing BMI (*n =* 1197 (22.8%)) values were replaced with ≥18.5 to <23 (normal).

The following accompanying symptoms and past medical histories were recorded: diabetes mellitus, hypertension, heart failure, chronic heart disease, chronic obstructive pulmonary disease, chronic kidney disease, cancer, chronic liver disease, rheumatic or autoimmune disease, and dementia.

### 2.7. Statistical Analyses

The general characteristics of the severe and mild groups were compared using the chi-square or Fisher’s exact test for categorical variables and the independent *t* test for continuous variables.

To analyze the hazard ratios (HRs) with 95% confidence intervals (CIs) for clinical symptoms related to death, a Cox proportional hazard regression model was used. In this analysis, crude and adjusted models (adjusted age, sex, obesity, systolic blood pressure, diastolic blood pressure, heart rate, body temperature, diabetes, hypertension, heart failure, chronic heart disease, asthma, chronic obstructive pulmonary disease, chronic kidney disease, cancer, chronic liver disease, rheumatic or autoimmune disease, and dementia) were calculated.

To analyze the odds ratios (ORs) of clinical symptoms related to morbidity, a logistic regression model was used. In this analysis, crude and adjusted models (adjusted for the variables above) were calculated.

Two-tailed analyses were performed, and significance was defined as a *p* value less than 0.05. SAS version 9.4 (SAS Institute Inc., Cary, NC, USA) was used for the statistical analyses.

## 3. Results

Of the COVID-19 patients, 15.4% (808/5253) and 84.6% (4445/5253) were classified as having severe and mild morbidity, respectively (Table 1). Age; sex; systolic blood pressure; diastolic blood pressure; heart rate; and past medical histories of diabetes, hypertension, heart failure, chronic heart disease, asthma, chronic obstructive pulmonary disease (COPD), chronic kidney disease, any cancer, and chronic liver disease were significantly different between severe and mild COVID-19 patients (all *p* < 0.05). The severe COVID-19 group had higher rates of respiratory symptoms including cough, sputum production, sore throat, and shortness of breath; gastrointestinal symptoms including vomiting and nausea and diarrhea; general symptoms including fever and fatigue and malaise; and neurologic symptoms including altered mental status or confusion (all *p* < 0.05). Headache and rhinorrhea were more common in mild COVID-19 patients than in severe COVID-19 patients (6.2% vs. 10.9%, *p* < 0.001 for rhinorrhea and 13.6% vs. 17.3%, *p* = 0.011 for headache).

Regarding each initial symptom, rhinorrhea, shortness of breath, fever, headache, and altered mental status or confusion were associated with mortality due to COVID-19 (Table 2). Shortness of breath, fever, and altered mental status or confusion were associated with 3.33- (95% CI = 2.52–4.40, *p* < 0.001), 1.50- (95% CI = 1.05–2.15), and 3.89-fold (95% CI = 2.40–6.31) increased HRs for mortality (Table 2). On the other hand, rhinorrhea and headache were associated with 0.41- (95% CI = 0.18–0.92) and 0.54-fold (0.31–0.96) decreased HRs for mortality. The groups of symptoms were classified as respiratory, gastrointestinal, general, and neurologic symptoms, and the association of each group of symptoms with mortality was analyzed. The respiratory symptom score was associated with a 1.18-fold increased HR for mortality (95% CI = 1.05–1.33, *p* = 0.004). All the groups of symptoms were fully adjusted to construct a full insertion model (adjusted respiratory, gastrointestinal, general, and neurologic symptoms). In this full insertion model, the respiratory symptom score was also associated with high mortality (adjusted HR = 1.17, 95% CI = 1.04–1.32, *p* = 0.008).

Several initial symptoms were related to high morbidity; the adjusted ORs were 1.56 (95% CI = 1.30–1.87) for cough, 1.52 (95% CI = 1.26–1.84) for sputum, 7.17 (95% CI = 5.75–8.95) for shortness of breath, 1.56 (1.09–2.22) for vomiting and nausea, 1.41 (95% CI = 1.06–1.89) for diarrhea, 2.38 (95% CI = 1.87–3.05) for fever, and 8.02 (95% CI = 3.13–20.53) for altered mental status or confusion (Table 3). According to the groups of symptoms, respiratory, gastrointestinal, and general symptoms were associated with high morbidity (adjusted OR = 1.45, 95% CI = 1.34–1.57, *p* < 0.001 for respiratory symptoms; adjusted OR = 1.40, 95% CI = 1.13–1.72, *p* = 0.002 for gastrointestinal symptoms; and adjusted OR = 1.53, 95% CI = 1.32–1.76, *p* < 0.001 for general symptoms). In the full insertion model, respiratory and general symptom scores were associated with 1.41- (95% CI = 1.30–1.53) and 1.37-fold (95% CI = 1.18–1.59) increased odds of morbidity.

## 4. Discussion

Respiratory symptoms were associated with high mortality due to COVID-19 in the present study. In addition to respiratory symptoms, general symptoms were related to high morbidity associated with COVID-19. Each initial symptom was analyzed to determine its relationship with morbidity and mortality in COVID-19 patients in this study. Although prior studies suggested that the severity and prognosis of COVID-19 was associated with respiratory or nonrespiratory symptoms, there has been little concern regarding other categories of initial symptoms with COVID-19-related morbidity and mortality.

Respiratory symptoms have been reported to be associated with the severe disease course of COVID-19 and accompanied by a high inflammatory marker indexes [12,13,14,15]. Severe COVID-19 has been reported to be characterized by hyperinflammation with excessive cytokine production, which results in lymphopenia and IL-6-mediated inhibition of HLA-DR expression [12]. Mortality in patients with COVID-19 has been associated with both pulmonary and systemic inflammation, which cause multiorgan dysfunction [13]. In nonsurviving patients, the rates of dyspnea (62% vs. 31%), chest discomfort (49% vs. 30%), and consciousness disorder (22% vs. 1%) were more common than those in recovered patients in a previous patient series [13]. The symptoms of dyspnea, chest pain, cough, lymphopenia and inflammatory marker elevation were more common in severe COVID-19 patients than in regular patients [15].

Unlike respiratory symptoms, gastric and neurologic symptoms were not related to COVID-19-related morbidity and mortality in the present study. In full insertion model, gastrointestinal symptoms were not related with both mortality and morbidity of COVID-19. In symptom score model, gastrointestinal symptoms were associated with morbidity, but not mortality of COVID-19. The contribution of gastric symptoms to the mortality of disease may have been masked by other symptoms (fever and dyspnea) in this study. Extrapulmonary symptoms of COVID-19, including gastrointestinal and neurologic, have been reported [16,17]. Coronaviruses has been suggested to be transmitted enterically because of adhesion to angiotensin-converting enzyme 2 cell receptors [18,19]. A retrospective study reported that diarrhea was associated with a mild disease course of COVID-19 [11]. The authors found that patients with diarrhea had lower rates of systemic steroid use and ECMO therapy, milder clinical courses without intensive care unit admission, and lower rates of septic shock and acute respiratory distress syndrome than nondiarrhea patients [11]. Other coronavirus infections causing severe acute respiratory syndrome (SARS) were associated with gastrointestinal symptoms and watery diarrhea in approximately 38.4% of patients [20]. However, most diarrhea is self-limiting and causes minimal disturbance of the intestinal architecture [20]. Another cross-sectional study reported a longer duration from the onset of gastric symptoms to admission in patients with gastric symptoms than in those without gastric symptoms (9.0 days vs. 7.3 days) [19]. Because oxygen therapy and intensive care unit admission are generally due to respiratory distress, intensive care could be delayed in patients with gastric symptoms but no or mild respiratory symptoms. On the other hand, gastric symptoms could be related to severe COVID-19 due to infection by the mutated form of SARS-CoV-2 [21]. A total of 23.0% (17/74) of patients with gastric symptoms presented with severe disease; this number was higher than the number of patients without gastric symptoms [21]. A considerable portion of these patients also experienced fever (39.2%), fatigue (31.1%), dyspnea (10.8%), and headache (21.6%) [21].

SARS-CoV-2 can invade the central nervous system and manifest as neurologic symptoms such as headaches [22]. In addition, SARS-CoV-2 can spread from the mechanoreceptors or chemoreceptors of respiratory tracts to the medullary cardiorespiratory center via a synapse-connected route [22]. This type of viral invasion has been suggested to contribute to respiratory distress in COVID-19 patients [22]. Thus, it can be presumed that initial respiratory symptom cannot exclude neural invasion in COVID-19 patients.

This study described several initial symptoms of COVID-19 that were associated with mortality and/or morbidity. A few limitations should be considered when interpreting the current results. Inflammatory markers and pulmonary function could not be accessed in this study. This study included extrapulmonary symptoms of vomiting/nausea, diarrhea, headache, and altered mental status or confusion. However, a number of extrapulmonary symptoms, such as cardiac, hepatic, renal, olfactory/gustatory, cutaneous, and hematological symptoms, were not included due to lack of information in this cohort. Some of these extrapulmonary symptoms, such as cardiac involvement, acute kidney injury, and hematologic complications, were related to a poor prognosis of COVID-19 [16], while past medical histories of hypertension, heart failure, chronic heart disease, chronic kidney disease, cancer, and chronic liver disease were adjusted as covariates. The time duration from the onset of initial symptom to the hospitalization could not be accessed in the present study. In addition, medications and/or specific treatment procedures were not considered in the current study. Despite these limitations, the results of the present study could be clinically valuable for triaging COVID-19 patients based on initial symptoms and developing treatment strategies.

## 5. Conclusions

Initial respiratory symptoms of COVID-19 were associated with high morbidity and mortality. The general symptom of fever was related to high mortality due to COVID-19, while other gastric and neurologic symptoms were not related to COVID-19-related morbidity and mortality.

## Figures and Tables

**Table 1:** General characteristics of the participants with COVID-19 according to severity of morbidity upon hospital admission.

Characteristics	The Participants with COVID-19		
	Severe	Mild	*p*-Value
Total participants (*n,* %)	808 (100.0)	4445 (100.0)	
Age (years old) (*n,* %)			<0.001 *
0–9	0 (0.0)	66 (1.5)	
10–19	2 (0.3)	192 (4.3)	
20–29	21 (2.6)	976 (22.0)	
30–39	16 (2.0)	503 (11.3)	
40–49	36 (4.5)	643 (14.5)	
50–59	136 (16.8)	932 (21.0)	
60–69	191 (23.6)	675 (15.2)	
70–79	214 (26.5)	327 (7.4)	
80+	192 (23.8)	131 (3.0)	
Sex (*n,* %)			<0.001 *
Male	380 (47.0)	1796 (40.4)	
Female	428 (53.0)	2649 (59.6)	
Obesity ‡ (*n,* %)			0.066
Underweight	34 (4.2)	213 (4.8)	
Normal	430 (53.2)	2465 (55.5)	
Overweight	137 (17.0)	815 (18.3)	
Obese I	178 (22.0)	787 (17.7)	
Obese II	29 (3.6)	165 (3.7)	
Systolic blood pressure (*n,* %)			<0.001 *
<120 mmHg	166 (20.5)	1077 (24.2)	
120–129 mmHg	167 (20.7)	1042 (23.4)	
130–139 mmHg	141 (17.5)	871 (19.6)	
140–159 mmHg	223 (27.6)	1080 (24.3)	
≥160 mmHg	111 (13.7)	375 (8.4)	
Diastolic blood pressure (*n,* %)			0.024 *
<80 mmHg	338 (41.8)	1615 (36.3)	
80–89 mmHg	253 (31.3)	1577 (35.5)	
90–99 mmHg	146 (18.1)	850 (19.1)	
≥100 mmHg	71 (8.8)	403 (9.1)	
Heart rate (mean, SD)	87.28 (17.1)	85.29 (14.5)	0.002 †
Temperature (mean, SD)	36.91 (0.5)	36.87 (0.5)	0.564
Past medical history			
Diabetes mellitus (*n,* %)	229 (28.3)	447 (10.1)	<0.001 *
Hypertension (*n,* %)	382 (47.3)	783 (17.6)	<0.001 *
Heart failure (*n,* %)	35 (4.3)	24 (0.5)	<0.001 *
Chronic heart disease (*n,* %)	68 (8.4)	109 (2.5)	<0.001 *
Asthma (*n,* %)	30 (3.7)	94 (2.1)	0.006 *
COPD (*n,* %)	20 (2.5)	20 (0.5)	<0.001 *
Chronic kidney disease (*n,* %)	32 (4.0)	22 (0.5)	<0.001 *
Any cancer (*n,* %)	44 (5.5)	101 (2.3)	<0.001 *
Chronic liver disease (*n,* %)	22 (2.7)	59 (1.3)	0.003 *
Rheumatic or autoimmune disease (*n,* %)	9 (1.1)	29 (0.7)	0.155
Dementia (*n,* %)	215 (4.2)	8 (6.5)	0.218
Clinical initial symptoms			
Respiratory symptoms			
Cough (*n,* %)	383 (47.4)	1812 (40.8)	<0.001 *
Sputum (*n,* %)	283 (35.0)	1222 (27.5)	<0.001 *
Sore throat (*n,* %)	78 (9.7)	723 (16.3)	<0.001 *
Rhinorrhea (*n,* %)	50 (6.2)	483 (10.9)	<0.001 *
Shortness of breath (*n,* %)	325 (40.2)	302 (6.8)	<0.001 *
Gastrointestinal symptoms			
Vomiting and nausea (*n,* %)	62 (7.7)	178 (4.0)	<0.001 *
Diarrhea (*n,* %)	87 (10.8)	375 (8.4)	0.031 *
General symptoms			
Fever (*n,* %)	336 (41.6)	906 (20.4)	<0.001 *
Myalgia (*n,* %)	141 (17.5)	718 (16.2)	0.359
Fatigue and malaise (*n,* %)	59 (7.3)	170 (3.8)	<0.001 *
Neurologic symptoms			
Headache (*n,* %)	110 (13.6)	767 (17.3)	0.011 *
Alteration of confusion (*n,* %)	27 (3.3)	8 (0.2)	<0.001 *
Death (*n,* %)	241 (29.8)	0 (0.0)	<0.001 *

* Chi-square or Fisher’s exact test. Significance at *p* < 0.05. † Independent *t* test. Significance at *p* < 0.05. ‡ Obesity (BMI, body mass index, kg/m^2^) was categorized as <18.5 (underweight), ≥18.5 to <23 (normal), ≥23 to <25 (overweight), ≥25 to <30 (obese I), and ≥30 (obese II).

**Table 2:** Crude and adjusted hazard ratios (95% confidence interval) for death in clinical findings at the time of hospitalization.

Characteristics	HRs for Death			
	Crude	*p*-Value	Adjusted †	*p*-Value
**Symptom**				
Cough	0.66 (0.50–0.86)	0.002 *	0.90 (0.67–1.20)	0.466
Sputum	1.02 (0.77–1.34)	0.904	1.19 (0.88–1.60)	0.256
Sore throat	0.31 (0.18–0.55)	<0.001 *	0.81 (0.46–1.43)	0.466
Rhinorrhea	0.22 (0.10–0.50)	<0.001 *	0.41 (0.18–0.92)	0.036 *
Shortness of breath	6.39 (4.96–8.25)	<0.001 *	3.33 (2.52–4.40)	<0.001 *
Vomiting and nausea	1.35 (0.81–2.24)	0.252	1.01 (0.60–1.69)	0.977
Diarrhea	0.79 (0.49–1.28)	0.342	0.94 (0.57–1.54)	0.803
Fever	1.97 (1.52–2.55)	<0.001 *	1.50 (1.05–2.15)	0.024 *
Myalgia	0.46 (0.29–0.72)	<0.001 *	0.74 (0.47–1.18)	0.210
Fatigue and malaise	1.63 (0.99–2.66)	0.054	1.04 (0.61–1.75)	0.895
Headache	0.28 (0.16–0.48)	<0.001 *	0.54 (0.31–0.96)	0.036 *
Alteration of confusion	20.75 (13.46–31.98)	<0.001 *	3.89 (2.40–6.31)	<0.001 *
**Symptom score**				
Respiratory symptom	1.06 (0.95–1.18)	0.320	1.18 (1.05–1.33)	0.004 *
Gastrointestinal symptom	0.99 (0.71–1.38)	0.947	0.97 (0.69–1.37)	0.878
General symptom	1.19 (1.00–1.43)	0.049 *	1.09 (0.87–1.38)	0.442
Neurologic symptom	0.82 (0.58–1.17)	0.275	1.26 (0.88–1.80)	0.217
**Full insertion model ‡**				
Respiratory symptom	1.05 (0.94–1.18)	0.393	1.17 (1.04–1.32)	0.008 *
Gastrointestinal symptom	0.96 (0.69–1.35)	0.830	0.92 (0.65–1.31)	0.652
General symptom	1.22 (1.01–1.46)	0.039 *	1.02 (0.80–1.29)	0.884
Neurologic symptom	0.75 (0.52–1.08)	0.121	1.18 (0.81–1.71)	0.388

* Cox proportional hazard regression model, Significance at *p* < 0.05. † The model was adjusted for age, sex, obesity, systolic blood pressure, diastolic blood pressure, heart rate, temperature, diabetes, hypertension, heart failure, chronic heart disease, asthma, chronic obstructive pulmonary disease, chronic kidney disease, cancer, chronic liver disease, rheumatic or autoimmune disease, and dementia. ‡ Adjusted respiratory, gastrointestinal, general, and neurologic symptoms

**Table 3:** Crude and adjusted odds ratios (95% confidence interval) for severe morbidity during the admission.

Characteristics	Ors For Severe Morbidity			
	Crude	*p*-Value	Adjusted †	*p*-Value
**Symptom**				
Cough	1.31 (1.13–1.52)	<0.001 *	1.56 (1.30–1.87)	<0.001 *
Sputum	1.42 (1.21–1.67)	<0.001 *	1.52 (1.26–1.84)	<0.001 *
Sore throat	0.55 (0.43–0.70)	<0.001 *	0.87 (0.66–1.15)	0.341
Rhinorrhea	0.54 (0.40–0.73)	<0.001 *	0.77 (0.54–1.08)	0.131
Shortness of breath	9.23 (7.69–11.08)	<0.001 *	7.17 (5.75–8.95)	<0.001 *
Vomiting and nausea	1.99 (1.48–2.69)	<0.001 *	1.56 (1.09–2.22)	0.015 *
Diarrhea	1.31 (1.02–1.68)	0.032 *	1.41 (1.06–1.89)	0.020 *
Fever	2.78 (2.38–3.26)	<0.001 *	2.38 (1.87–3.05)	<0.001 *
Myalgia	1.10 (0.90–1.34)	0.359	1.21 (0.96–1.52)	0.109
Fatigue and malaise	1.98 (1.46–2.69)	<0.001 *	1.45 (0.99–2.12)	0.056
Headache	0.76 (0.61–0.94)	0.011 *	1.00 (0.78–1.28)	0.997
Alteration of confusion	19.17 (8.68–42.36)	<0.001 *	8.02 (3.13–20.53)	<0.001 *
**Symptom score**				
Respiratory symptom	1.32 (1.24–1.41)	<0.001 *	1.45 (1.34–1.57)	<0.001 *
Gastrointestinal symptom	1.46 (1.22–1.75)	<0.001 *	1.40 (1.13–1.72)	0.002 *
General symptom	1.71 (1.54–1.90)	<0.001 *	1.53 (1.32–1.76)	<0.001 *
Neurologic symptom	0.97 (0.79–1.18)	0.744	1.18 (0.94–1.48)	0.165
**Full insertion model ‡**				
Respiratory symptom	1.26 (1.18–1.35)	<0.001 *	1.41 (1.30–1.53)	<0.001 *
Gastrointestinal symptom	1.26 (1.04–1.52)	0.017 *	1.22 (0.98–1.52)	0.069
General symptom	1.62 (1.45–1.80)	<0.001 *	1.37 (1.18–1.59)	<0.001 *
Neurologic symptom	0.69 (0.56–0.85)	0.001 *	0.87 (0.68–1.11)	0.274

* Cox proportional hazard regression model, Significance at *p* < 0.05. † The model was adjusted for age, sex, obesity, systolic blood pressure, diastolic blood pressure, heart rate, temperature, diabetes, hypertension, heart failure, chronic heart disease, asthma, chronic obstructive pulmonary disease, chronic kidney disease, cancer, chronic liver disease, rheumatic or autoimmune disease, and dementia. ‡ Adjusted respiratory, gastrointestinal, general, and neurologic symptoms

## Data Availability

Releasing of the data by the researcher is not allowed legally. All data are available from the database of the Korea Centers for Disease Control and Prevention. The Korea Centers for Disease Control and Prevention allows data access, at a particular cost, for any researcher who promises to follow the research ethics. Data of this article can be downloaded from the website after promising to follow the research ethics.
